# Abnormal Haemodynamic Flow Patterns in Bicuspid *Pulmonary* Valve Disease

**DOI:** 10.3389/fphys.2017.00374

**Published:** 2017-05-31

**Authors:** Malenka M. Bissell, Margaret Loudon, Stefan Neubauer, Saul G. Myerson

**Affiliations:** Division of Cardiovascular Medicine, Radcliffe Department of Medicine, University of Oxford Centre for Clinical Magnetic Resonance ResearchOxford, United Kingdom

**Keywords:** helical flow, 4D flow MRI, bicuspid valve disease, dilation, humans

## Abstract

Abnormal flow patterns in the aortas of those with bicuspid aortic valves (BAVs) are increasingly recognized as important in the pathogenesis of aortic dilatation but pulmonary flow patterns in bicuspid *pulmonary* valves have not been studied. Bicuspid *pulmonary* valve disease is rare and a small numbers of case reports describe concomitant pulmonary artery dilation similar to the dilation of the ascending aorta, which is often seen in BAVs disease. We examined three cases of bicuspid *pulmonary* valve disease, 10 healthy volunteers and 10 patients with BAV disease but a tricuspid *pulmonary* valve. All participants underwent anatomical and functional imaging of the pulmonary valve, pulmonary artery, and right ventricle as well as advanced time-resolved 3-dimensional cardiac magnetic resonance imaging (4D flow) to assess the flow pattern in the pulmonary artery. All patients with a bicuspid *pulmonary* valve had pulmonary artery dilation and showed distinct helical flow abnormalities with increased rotational flow and increased flow displacement compared to a mild left-handed flow pattern in the healthy volunteers. Additionally, there was marked asymmetry seen in the systolic wall shear stress (WSS) pattern, with the highest values in the anterior wall of the pulmonary artery. In comparison, patients with a BAV but a tricuspid *pulmonary* valve had normal flow patterns in the pulmonary artery. These haemodynamic findings are similar to recent studies in bicuspid *aortic* disease, and suggest the importance of flow patterns in the pathophysiology of vessel dilation in both aortic and pulmonary bicuspid valve disease.

## Introduction

Recent advances in cardiovascular magnetic resonance imaging have altered the understanding of the pathophysiology of aortic dilation in bicuspid *aortic* valve (BAV) disease. 4D flow magnetic resonance imaging (4D flow MRI) allows visualization and quantification in all major blood vessels in a 3D image, time resolved over the cardiac cycle. The acquisition slab over the area of interest includes time resolved velocity encoding images in three directions and a time resolved magnitude image per slice. These images are then corrected for Maxwell effects, aliasing and eddy currents and then reconstructed for visualization. For analysis, analysis planes are placed perpendicular to the vessel of interest and hemodynamic flow changes are quantified, such as flow angle and flow displacement, calculating how much the flow jet deviates from the midline of the vessel, rotational flow (circulation, an integral of vorticity), and wall shear stress (WSS) estimations based on the interpolation of local velocity derivatives. These visualization and quantification methods have been applied to BAV disease and shown, that the majority of patients exhibit a marked right-handed helical flow pattern (Hope et al., 2011; Barker et al., 2012; Bissell et al., 2013; Meierhofer et al., 2013). There findings have led to the hypothesis that haemodynamic flow disturbances, in the form of increased flow angle and flow displacement (leading to increased helical flow and thereby increased asymmetrical WSS, play a major part in the development of aortic dilation (Hope et al., 2011; Barker et al., 2012; Bissell et al., 2013; Meierhofer et al., 2013). The concept that increased WSS contributes to an aortopathy is further supported by a recent study examining histopathological changes in excised BAV aortas. Changes such as reduced elastin were only present in areas with increased WSS, but not in areas with normal WSS as assessed with 4D flow MRI prior to aortic resection (Guzzardi et al., 2015).

If the observed flow changes are indeed caused by a bicuspid valve, we hypothesized that these flow changes may also be present in bicuspid *pulmonary* valve disease. However, isolated bicuspid *pulmonary* valve disease is rare with only few case reports in the literature. In 1955, Ford et al. reviewed the literature and found as few as 15 cases with a confirmed bicuspid *pulmonary* valve (Ford et al., 1956). Autopsy findings in the case described by Ford et al. already documented a markedly dilated pulmonary artery with normal arterial wall structure, a finding confirmed in later case reports (Jodocy et al., 2009; Vedanthan et al., 2009; Goda et al., 2012; Krauss et al., 2014). To date nothing is known about flow pattern in the pulmonary arteries in these isolated cases. The aim of this study was two-fold:

1. To assess whether helical flow patterns were present in the pulmonary artery in patients with a bicuspid *pulmonary* valve suggesting it is the bicuspid valve itself contributing to flow patterns. This would be done using 4D flow MRI to examine flow patterns in the pulmonary artery in patients with a bicuspid *pulmonary* valve compared to healthy volunteers.
2. To assess if BAV patients with morphological normal pulmonary valves also exhibit helical flow patterns in the pulmonary artery, suggesting other factors may be involved in the generation of helical flow patterns. Again, 4D flow MRI flow patterns in patients with a BAV compared to healthy volunteers will be compared.

## Materials and methods

Twenty-three prospectively enrolled participants underwent 4D flow MRI assessment including the assessment of flow patterns in the main pulmonary artery. This included three patients with a bicuspid *pulmonary* valve with a normal aortic valve, 10 age- and sex matched healthy volunteers (male, mean age 61 ± 9 years) and 10 age- and sex-matched patients with a BAV but normal tricuspid *pulmonary* valve (male, mean age 61 ± 8 years).

### Cardiovascular magnetic resonance (CMR) acquisition

Each subject underwent two CMR scans—one on a 1.5 Tesla system (Avanto, Siemens, Erlangen, Germany) for anatomical imaging; the second on a 3.0 Tesla system (Trio, Siemens, Erlangen, Germany) for 4D flow assessment, both using a 32-channel cardiac coil. All images were electrocardiogram (ECG)-gated. Steady-state free-precession (SSFP) cine sequences acquired during a single breath-hold were used for pulmonary artery dimension measurements, right ventricular volume assessment and pulmonary valve morphology. The velocity across the pulmonary valve was measured using through-plane phase contrast velocity mapping in an image slice placed perpendicular to the pulmonary artery, just above the valve tips. CMR42 (Circle Cardiovascular Imaging Inc., Calgary, Canada) was used for analysis of standard anatomical and velocity parameters.

### 4D flow MRI assessment

**Figure 1:**
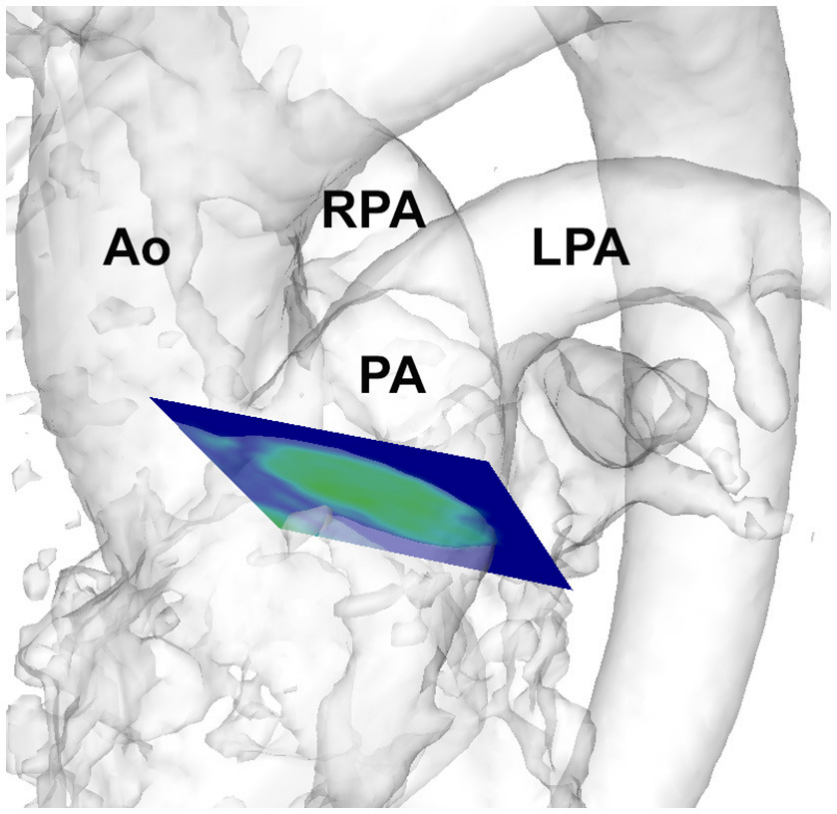
Depiction of analysis plane placement in the pulmonary artery in a healthy volunteer. Ao, aorta; PA, pulmonary artery; RPA, right pulmonary artery; LPA, left pulmonary artery.

Flow-sensitive gradient-echo pulse sequence CMR datasets were acquired with prospective ECG-gating during free-breathing, using a respiratory navigator. The image acquisition volume was in an oblique sagittal plane encompassing the thoracic aorta, main pulmonary artery and the proximal pulmonary branch arteries. Sequence parameters: echo time 2.5 ms, repetition time 5.1 ms, flip angle 7°, voxel size 2.0 × 1.7 × 2.2 mm^3^, temporal resolution 40 ms. The velocity encoding range was determined using the lowest non-aliasing velocity on scout measurements. Data acquisition lasted 10–15 min and the data was therefore collected from and averaged over hundreds of cardiac cycles. Dataset processing and WSS calculation were conducted with customized Matlab software Version R2010a (The MathWorks Inc., Natick, Massachusetts, USA) and EnSight Version 9.1.2 (CEI Inc., Apex, North Carolina, USA), as described previously (Frydrychowicz et al., 2007; Markl et al., 2007; Stalder et al., 2008; Barker et al., 2012). The 3-dimensional flow patterns were measured in a short axis slice through the main pulmonary artery (Figure 1). Measurements were averaged over systole in the acquired cardiac cycle (one time frame before and three after peak systolic flow) to mitigate measurement noise (Barker et al., 2012).

### 4D flow MRI quantification

Flow through a BAV has been shown to be highly abnormal with markedly accentuated helical flow (Hope et al., 2010). Helical flow is composed of a forward component (along the long axis of the aorta) and a rotational component (rotating around the long axis in a circumferential direction). The rotational component of helical flow can be quantified using the circulation measure, which is the integral of vorticity with respect to the cross-sectional area of the aorta (Farthing and Peronneau, 1979; Hess et al., 2013).

WSS was calculated using the 3-dimensional flow vector and magnitude data using the published analysis method by Stalder et al. (2008). WSS quantifies the shearing force of the moving blood against the vessel lumen using the viscosity of the blood, deformation tensor (which includes the velocity components and spatial dimensions as part of the three-directional velocity field of the acquired CMR data) and inward unit normal (which describes the direction toward the center of the vessel; Stalder et al., 2008). In the analysis plane, the vessel wall was manually traced for each time frame within the cardiac cycle to define the area for analysis. Systolic WSS was measured in eight anatomical positions within the pulmonary artery lumen.

The systolic flow angle was also calculated—this is defined as the angle between the line perpendicular to the short axis analysis plane and the instantaneous mean flow vector at peak systole (Entezari et al., 2013). Flow displacement was defined as the distance (in millimeters) from the vessel centroid to the velocity-weighted centroid (Mahadevia et al., 2014).

Student *t*-test was used for statistical comparison where appropriate. A *p* < 0.05 was considered significant. Mean values were reported ± one standard deviation. For comparison with the individual bicuspid *pulmonary* valve patients, we also reported the minimum and maximum values of healthy volunteers to indicate the range of these values normally seen in a healthy population sample.

This study was carried out in accordance with the recommendations of the West Berkshire ethics committee with written informed consent from all subjects. All subjects gave written informed consent in accordance with the Declaration of Helsinki. The protocol was approved by the West Berkshire ethics committee.

### Patient characteristics

Of the three patients with bicuspid *pulmonary* valves, patient 1 (69 years) was one of the first “blue” babies that underwent catheter balloon valvotomy of his *pulmonary* valve in London in 1949. He has not needed any intervention since. He was a non-smoker, overweight (BMI 35) and on no cardiovascular medications but hypertensive during the study visit (169/69 mmHg). He also suffered from gout.

Patient 2 (73 years) was diagnosed incidentally with a bicuspid *pulmonary* valve during a recent cardiovascular magnetic resonance exam. He was an ex-smoker, normotensive and on no cardiovascular medications.

Patient 3 (47 years) had more complex heart disease with a double outlet right ventricle and marked pulmonary stenosis. He was an ex-smoker, normotensive, and on no cardiovascular medications. He also suffered from gout.

BAV disease patients had isolated valve disease with no other cardiovascular problems such as coronary artery disease or coarctation of the aorta. The age range was 47–72 years. The patients were normotensive, 4 patients were on blood pressure lowering agents, 1 participants still smoked, and 4 participants were ex-smokers. All participants had normal left ventricular function (ejection fraction 57–70%). Two patients suffered from gastroesophageal reflux.

All healthy volunteers were free from cardiovascular disease or any other non-cardiac disease. The age range was 47–75 years. They were normotensive and not actively smoking.

## Results

### Bicuspid *Pulmonary* valve disease

#### Pulmonary valve and right ventricular function

Patient 1 had a well-functioning bicuspid *pulmonary* valve (peak velocity across the pulmonary valve was 1.3 m/s with no regurgitation) and he had normal right ventricular (RV) function [ejection fraction 52%, RV end diastolic volume (RVEDD) 130 ml]. Patient 2 also had a well-functioning bicuspid *pulmonary* valve (peak velocity across the *pulmonary* valve was 1.6 m/s with no regurgitation) with normal right ventricular function (ejection fraction 56%, RVEDD 138 ml). Patient 3 had more complex heart disease with a double outlet right ventricle and marked pulmonary stenosis (narrowing of the valve, peak velocity 3.6 m/s with no regurgitation). All three patients were male and had a normal aortic valve with normal ascending aortic measurements. However, they all had a dilated pulmonary artery (4.5, 3.6, and 3.1 cm) compared with 2.6 ± 0.3 cm in the healthy volunteers.

#### 4D flow MRI quantification

**Figure 2:**
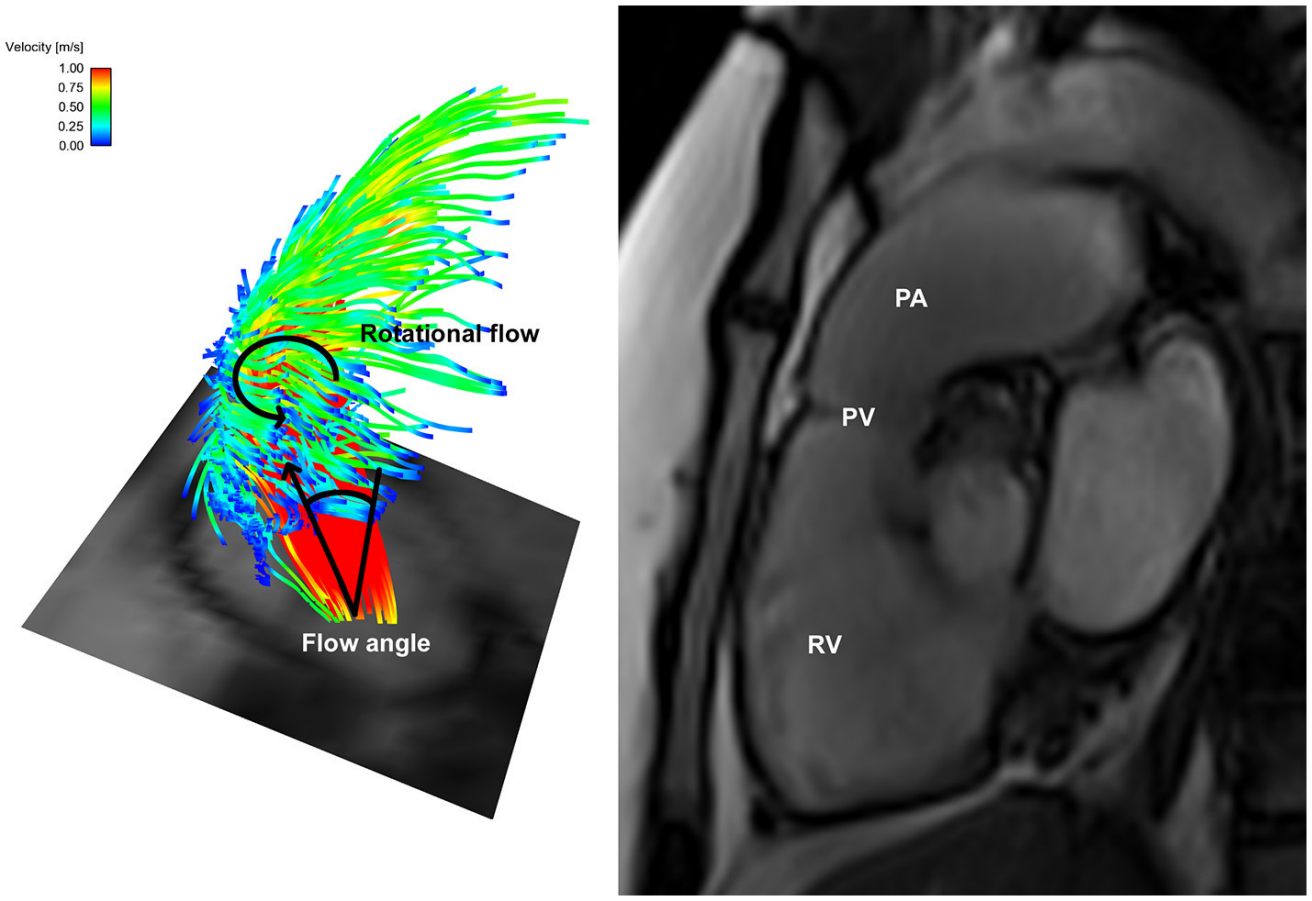
Helical flow pattern in bicuspid *pulmonary* valve disease patient 1—Left: Flow particle traces depiction showing marked right-handed helical flow pattern arising from the bicuspid pulmonary valve with an increased flow angle; Right: Dilated pulmonary artery in the same patient. RV, right ventricle; PV, pulmonary valve; PA, pulmonary artery.

**Figure 3:**
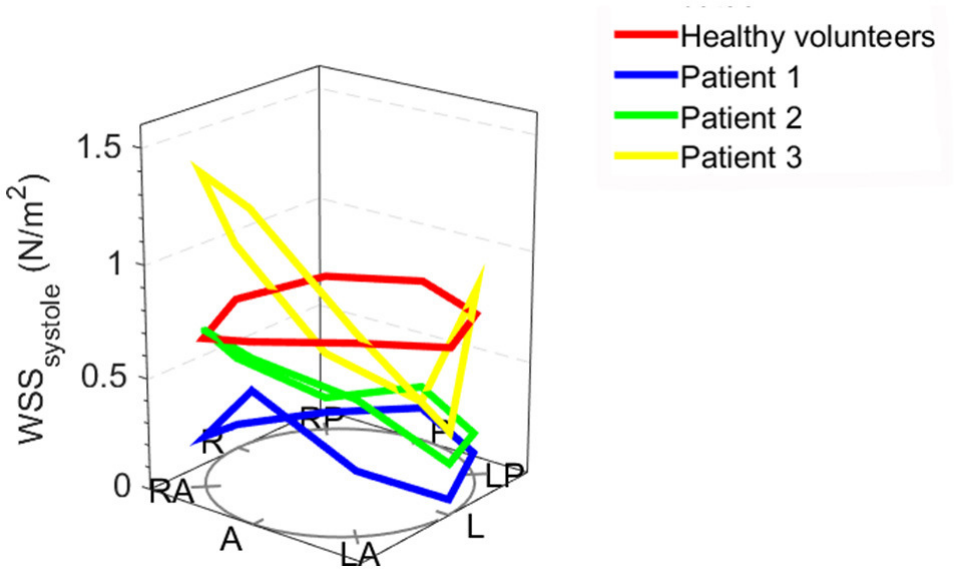
Systolic wall shear stress (WSS) in individual bicuspid *pulmonary* valve patients and the mean WSS values of healthy volunteers: the anatomical positions are shown at the bottom of the figure. A, anterior (outer curvature); LA, left anterior; L, left; LP, left posterior; P, posterior; RP, right posterior; R, right; RA, right anterior; the height of the dots indicates the WSS value.

When assessing the flow pattern in the pulmonary artery, patients 1 and 2 had a marked right-handed helical flow (rotational flow value 5.2 and 2.1 mm/m^2^; Figure 2) compared to a mild left-handed helical flow in healthy volunteers with a mean rotational flow of −1.2 ± 1.7 mm/m^2^. The 3rd patient had a marked left-handed helical flow (rotational flow value −9.5 mm/m^2^; Table 1). The flow angle with which the blood jet leaves the pulmonary valve was increased in 2 of the 3 patients (24.6, 9.2, and 17.4° vs. 9.8 ± 7.2° in healthy volunteers). Flow displacement was markedly increased in all 3 patients (15.1, 8.2, and 5.8 mm vs. 1.6 ± 0.7 mm in healthy volunteers). Comparing the WSS averaged over systole, the maximum WSS was lower in patient 1 and 2 (0.59 and 0.73 N/m^2^) than the healthy volunteers (0.88 ± 0.25 N/m^2^) and only elevated in patient 3 with a stenotic (narrowed) pulmonary valve (1.39 N/m^2^). However, there was a marked asymmetry seen in all three patients with highest WSS values in the anterior section with a marked anterior-posterior asymmetry (0.44, 0.48, and 1.18 N/m^2^) compared to healthy volunteers with negligible asymmetry (0.06 ± 0.12 N/m^2^; Figure 3).

**Table 1:** Wall shear stress quantification in bicuspid *pulmonary* valve disease compared to healthy volunteers.

	**Healthy volunteers**			**Bicuspid *pulmonary* valve**		
	**Mean**	**Minimum**	**Maximum**	**Patient 1**	**Patient 2**	**Patient 3**
Age in years	60.5	47	75	69	73	47
Rotational flow in mm/m^2^	−1.2	−4.1	1.4	**5.2**	**2.1**	**−9.5**
Flow angle in °	9.8	1.49	26.3	24.6	9.2	17.4
Displacement in mm	1.6	0.4	2.6	**15.1**	**8.2**	**5.8**
Pulmonary artery diameter in cm	2.6	2.2	3.1	**4.5**	**3.6**	3.1
Maximum Wall shear stress in N/m^2^	0.88	0.46	1.44	0.59	0.73	1.39
Wall shear stress ant-post asymmetry in N/m^2^	0.06	−0.09	0.29	**0.44**	**0.48**	**1.18**

*In bold are the patient values which are outside the healthy volunteer range*.

### The pulmonary artery in bicuspid *Aortic* valve disease

**Figure 4:**
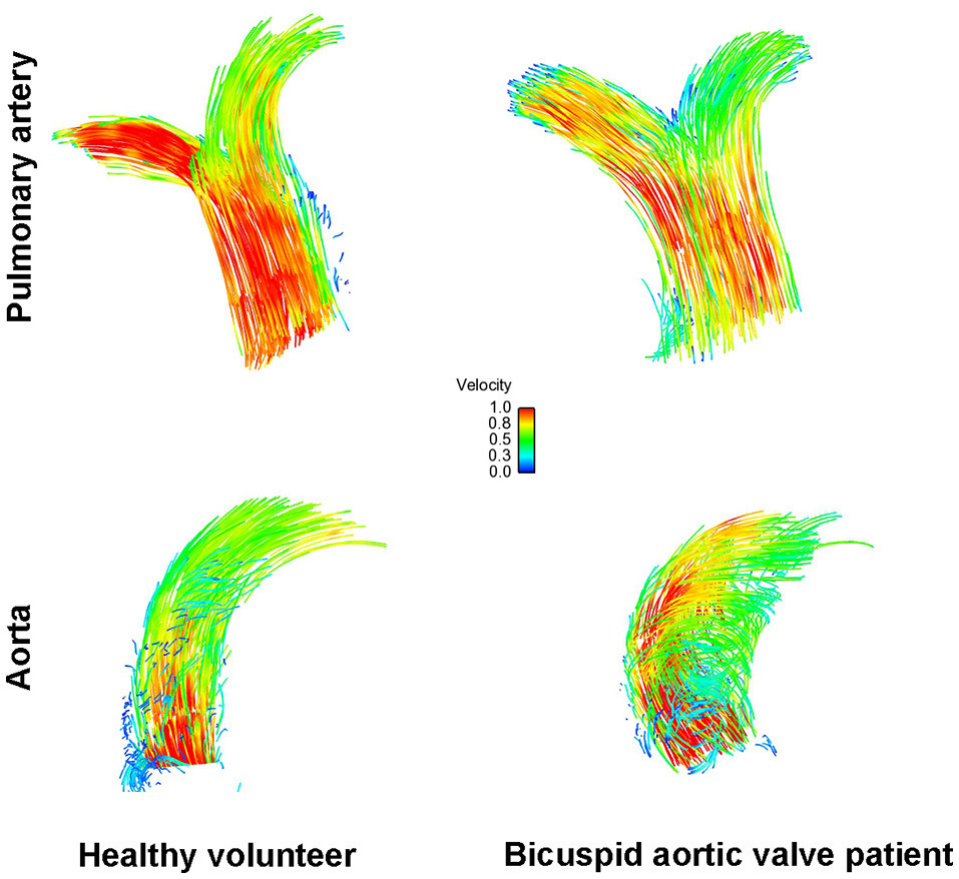
Flow particle traces depiction showing normal flow pattern in the pulmonary artery (top) and the aorta (bottom) in healthy volunteers (left) and in bicuspid *aortic* valve disease (right).

A recent echocardiographic publication suggested that the pulmonary artery may also be enlarged in patients with a BAV but normal trileaflet *pulmonary* valve (Kutty et al., 2010). We therefore also assessed the pulmonary artery in a sex- and age matched subgroup of our recently published BAV cohort (Bissell et al., 2013). There were no statistically significant differences however compared to healthy volunteers in pulmonary artery size (2.6 ± 0.2 cm vs. 2.6 ± 0.3 cm; *p* = 0.5), rotational flow (−0.6 ± 1.6 mm/m^2^ vs. −1.2 ± 1.7 mm/m^2^; *p* = 0.48), flow angle (9.4 ± 6.5° vs. 9.8 ± 7.2°; *p* = 0.39), displacement (1.9 ± 1.1 mm vs. 1.6 ± 0.7 mm; *p* = 0.41) and mean systolic WSS (0.61 ± 0.11 N/m^2^ vs. 0.88 ± 0.25 N/m^2^; *p* = 0.75; Figure 4).

## Discussion

We believe our study is the first to describe flow abnormalities in bicuspid *pulmonary* disease. Small numbers of case reports have described a dilated pulmonary artery in association with bicuspid *pulmonary* valves, raising the possibility of a similar underlying pathophysiology to that of BAV disease.

The advent of 4D flow imaging has shown abnormalities of flow patterns and WSS in the ascending aorta of patients with BAV disease. Several research units have described characteristic findings of increased wall shear in the anterior ascending aortic wall. It is not clear whether patients also have underlying aortic wall architectural abnormalities but a recent study demonstrated that changes in the aortic wall composition occur at sites of highest shear stress, suggesting flow is implicated in aortic dilatation and changes to the aortic wall composition may be a secondary phenomenon (Guzzardi et al., 2015).

In this study all three patients with a bicuspid *pulmonary* valve had a dilated pulmonary artery; furthermore all three patients also had rotational flow and flow displacement values outside the range seen in healthy volunteers. The observed WSS asymmetry, with increased values in the anterior pulmonary artery wall, are similar to these observed in the aorta of patients with a BAV (Bissell et al., 2013). These findings suggest the flow disturbances downstream of a bicuspid *pulmonary* valve are similar to those seen in BAV disease.

The morphology of the valve appears to be central to the development of abnormal flow patterns. In patients with a BAV but a tricuspid *pulmonary* valve these flow disturbances were seen only in the aorta but not the *pulmonary* arteries. The lack of flow abnormalities in the pulmonary arteries suggests it less likely that an intrinsic vessel wall abnormality (affecting the aorta and pulmonary artery) is the underlying cause for the observed abnormalities alone.

Blood into the pulmonary artery is ejected at much lower pressures than into the aorta which may explain the only mild effects of the helical flow pattern on WSS and wall dilation. While a bicuspid *pulmonary* valve may be clinically rare, these novel findings further underline the importance of haemodynamic flow disturbances in the pathophysiology of vessel dilation in bicuspid valve disease. To assess the prognostic value of 4D flow MRI and the clinical implication of our findings, longitudinal cohort studies of both bicuspid aortic and pulmonary valve disease are necessary to understand the degree of involvement haemodynamic changes have in vessel dilation and whether 4D flow MRI is a clinically useful imaging biomarker for predicting aortic growth rate.

### Limitations

As bicuspid *pulmonary* valve disease is rare, patient numbers were small. 4D flow MRI is a new imaging technique but measures such as WSS have been validated, even though the true WSS is likely to be higher than the measured value, due to limited spatial resolution and partial volume effects, as discussed previously (Markl et al., 2011). Furthermore, 4D flow MRI acquisition is averaged over hundreds of cycles. Therefore, we are unable to assess beat-to-beat variability. As patients are lying supine and are at rest during the imaging, the beat-to-beat variability is likely to be low.

## Author contributions

MB designed the work, completed the acquisition, analysis and interpretation of the work, drafted the work, approved the final version and agree to be accountable for all aspects of the work in ensuring that questions related to the accuracy or integrity of any part of the work are appropriately investigated and resolved. ML contributed to the design and acquisition of the work, revisited the work critically for important intellectual content, approved the final version and agree to be accountable for all aspects of the work in ensuring that questions related to the accuracy or integrity of any part of the work are appropriately investigated and resolved. SN and SM supervised the design of the study and interpretation of the work, revisited the work critically for important intellectual content, approved the final version and agree to be accountable for all aspects of the work in ensuring that questions related to the accuracy or integrity of any part of the work are appropriately investigated and resolved.

### Conflict of interest statement

The authors declare that the research was conducted in the absence of any commercial or financial relationships that could be construed as a potential conflict of interest.
